# Ginger as an anticolorectal cancer spice: A systematic review of in vitro to clinical evidence

**DOI:** 10.1002/fsn3.3153

**Published:** 2022-12-07

**Authors:** Seyed Mostafa Nachvak, Davood Soleimani, Mehali Rahimi, Ali Azizi, Mehdi Moradinazar, Mohammad Hossein Rouhani, Behrouz Halashi, Abbas Abbasi, Mahsa Miryan

**Affiliations:** ^1^ Nutritional Sciences Department, School of Nutritional Sciences and Food Technology Kermanshah University of Medical Sciences Kermanshah Iran; ^2^ Research Center of Oils and Fats Kermanshah University of Medical Sciences Kermanshah Iran; ^3^ Faculty of Medicine, Department of Nutrition Kermanshah University of Medical Sciences Kermanshah Iran; ^4^ Social Development and Health Promotion Research Center Kermanshah University of Medical Science Kermanshah Iran; ^5^ Food Security Research Center, Health Research Institute, Department of Clinical Nutrition, School of Nutrition and Food Science Isfahan University of Medical Sciences Isfahan Iran; ^6^ Department of Community Nutrition, School of Nutrition and Food Sciences, Kidney Diseases Research Center Isfahan University of Medical Sciences Isfahan Iran; ^7^ Student Research Committee Kermanshah University of Medical Science Kermanshah Iran

**Keywords:** anticancer plant, cell culture, colorectal cancer, ginger, systematic review

## Abstract

Ginger and its derivatives have been shown to be effective in the prevention and treatment of cancer. We undertook a systematic review to answer the question of whether ginger has a role in modifying the biomarkers of cancer in cell culture conditions and on colorectal cancer in randomized clinical trials. We performed a comprehensive search of the literature from Scopus, Embase, Web of Science, PubMed, Cochrane central register of controlled trials, and Cochrane database of systematic reviews. At first, all 12 papers studied the effect of ginger or its derivatives on cell culture conditions. The results of cell culture studies show that ginger has a powerful role in inducing apoptosis. In the second part, five studies of clinical trials were analyzed. By analyzing antitumor markers of clinical trials, ginger increased some anticancer markers but performed poorly in inducing some anticancer markers. This systematic review showed that the consumption of ginger extract has the potential to prevent and treat colorectal cancer but this ability is weak.

## Graphical abstract

This systematic review showed that the consumption of ginger extract has the potential to prevent and treatment of colorectal cancer but this ability is weak.

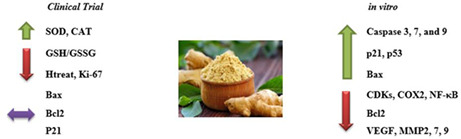

## 1 INTRODUCTION

Colorectal cancer (CRC) is a common malignant tumor with high morbidity and mortality worldwide. The most crucial cause of colon cancer can be considered aging and unhealthy lifestyle as well as genetic predisposition. Among unhealthy lifestyle factors, there is growing evidence about the roles of dietary components such as red meats, processed meats, and alcohol in the occurrence and development of CRC (Bent & Ko, 2004). On the other hand, some nutraceuticals in specific foods have benefits as well as few side effects in arresting cancer cells.

Ginger (*Zingiber officinale*) has been used worldwide as a spice, condiment, and medicinal remedy in many countries. Ginger and its active compounds, such as 6‐gingerol and 6‐shogaol, have shown beneficial biological effects including hepatoprotective, anti‐inflammation, antioxidation, and anticancer activities (Kim et al., 2014; Samadi et al., 2022). In many in vitro studies, it has been reported that ginger has a high ability to induce apoptosis in a variety of cancer cells (Arablou et al., 2014; Shidfar et al., 2015). One research has shown that ginger components such as 6‐ and 10‐gingerols have a beneficial role in the treatment of cervical cancer (Zhang et al., 2017). 6‐gingerol has inhibited cell proliferation, induced apoptosis, and blocked G1 cell‐cycle arrest in human colorectal cancer cells (Lee et al., 2008). In addition, it can induce apoptosis in human colorectal carcinoma cells through the activation of caspases (cysteine‐aspartic proteases, cysteine aspartate, or cysteine‐dependent aspartate‐directed proteases) and the production of reactive oxygen species (Lee et al., 2008). Several studies have shown that gingerol modulates a variety of cell signaling pathways linked to cancer, including nuclear factors (NF‐κB), signal transducer and activator of transcription 3 (STAT3), activator protein‐1 (AP‐1), β‐catenin, epidermal growth factor receptor (EGFR), vascular endothelial growth factor receptor (VEGFR), mitogen‐activated protein kinases (MAPK), and pro‐inflammatory mediators such as tumor necrosis factor (TNF‐α) and cyclooxygenase‐2 (COX‐2) (Jemal et al., 2009; Ling et al., 2010). In some studies, it has been also shown that the anticancer activity of ginger extract is significantly higher than curcumin in brain cancer cells (Ramachandran, Lollett, et al., 2015).

Since the cancer cell line models are a very useful diagnostic tool for the diagnosis of cancer at the starting point, most studies investigated the anticancer role of ginger in cell culture (Mirabelli et al., 2019). Questions in this study are as follows: Does ginger have a role in modifying the biomarkers of cancer in cell culture conditions? Does ginger have a role in modifying the biomarkers in colorectal cancer in clinical trials? At the end of this study, we compared the changes in cancer biomarkers in both conditions.

## 2 METHODS AND MATERIALS

### 2.1 Study design

This systematic review was performed by the preferred reporting items of systematic reviews and meta‐analyses (PRISMA) statement (Liberati et al., 2009). This systematic review was registered in the International Prospective Register of Systematic Reviews (PROSPERO) in 2022 (ID:CRD42022369388). Briefly, the aim is to identify the anticancer effects of ginger in cancer cell line models and clinical trials.

### 2.2 Search strategy

We systematically searched the Scopus, Embase, Web of Science, PubMed, Cochrane central register of controlled trials (CCTR), and Cochrane database of systematic reviews (CDSR) databases. Keywords for search in these databases were included:

1. For the first question: (“Ginger”[MeSH Terms] OR “Gingerol” OR “Shogaol”) AND (“Carcinogenesis”[MeSH Terms] OR “Carcinogenesis” OR “Cell Transformation” OR Neoplasms) OR (“Tumor”[MeSH Terms] OR “Cell Line”[MeSH Terms] OR “Cell Cultures” OR “Tumor Cell Lines” OR “Tumor Cell Line”)
2. For the second question: (“Ginger”[MeSH Terms] OR “Gingerol” OR “Shogaol”) AND (“Colorectal Neoplasms” [MeSH Terms] OR “Colorectal Tumors” OR “Colorectal Cancers” OR “Colorectal Carcinoma”) (“Clinical Trial [Publication Type]” [MeSH Terms] OR “Clinical Trial, Phase I” OR “Clinical Trial, Phase II” OR “Clinical Trial, Phase III” OR “Clinical Trial, Phase IV” OR “Controlled Clinical Trial” OR “Randomized Controlled Trial”).

The study was complemented by a search for these keywords in the databases mentioned above for eligible articles and email correspondences with authors for additional data where relevant. We included all cell cultures and randomized clinical trials that had studied the anticancer effect of ginger in human or human cancer cells.

### 2.3 Study selection

Study selection started with the removal of duplicates, followed by titles and abstracts to answer our question screening by two independent reviewers. To avoid bias, they were blinded to the names, qualifications, or institutional affiliations of the study authors. The full text of studies meeting inclusion criteria was retrieved and screened to determine eligibility by two reviewers. Following the assessment of methodological quality, two researchers extracted data using a purpose‐designed data extraction form and independently summarized what they considered to be the most important results from each study. These summaries were compared, and then any differences of opinion were resolved by discussion and consultation with a third reviewer. Any further calculations on study data considered necessary were conducted by the first reviewer and checked by the second reviewer. If the two authors failed to reach a consensus, the third author was involved in making a final decision.

### 2.4 Eligibility criteria

We included studies in this review if they were conducted on the anticancer effects of ginger at the cell culture condition and randomized clinical trials (RCT) that were published in the English language. Also, the studies included in this paper are clinical trials involving human participants with normal and increased risk of colorectal cancer. Exclusion criteria were as follows: articles that did not interfere with Ginger; articles that do not investigate cancer in cell culture and do not involve human participants with colorectal cancer; and articles that do not investigate the role of ginger in preventing and treating cancer.

### 2.5 Methodological quality assessment

The Cochrane risk‐of‐bias tool for randomized trials was used to assess the quality of the studies included in this systematic review. This tool consists of five bias domains, including selection bias (random sequence generation and allocation concealment), performance bias (blinding of participants and personnel), detection bias (blinding of outcome assessment), attrition bias (incomplete data outcome), and reporting bias (selective outcome reporting). We categorized clinical trial studies as Yes (low risk of bias), No (high risk of bias), or Unclear for each domain. Finally, the overall quality of the studies was categorized into weak, fair, or good, if <3, 3, or ≥4 domains were rated as low risk, respectively (Higgins et al., 2019; Raeisi‐Dehkordi et al., 2019).

### 2.6 Data extraction

In the present study, we analyzed several factors to evaluate the effect of ginger in preventing or treating cancer. One of the most important and key pathways to understanding the anticancer role of ginger is to study apoptosis. Therefore, we analyzed caspases, B‐cell lymphoma 2 (BCL2), BCL2‐associated X (BAX), and p21 to investigate the role of ginger in inducing apoptosis. Matrix metalloproteinases (MMPs) were analyzed to investigate the role of ginger in the proliferative factors. Also, to investigate the anti‐inflammatory effect of ginger, we analyzed the role of ginger in reducing inflammatory factors and enzymes involved in the inflammatory process such as cyclooxygenases (COXs), 5‐lipoxygenase (5‐LOX), 12‐LOX, 15‐LOX‐2, prostaglandin E2 (PGE2), and 15‐hydroxyeicosatetraenoic acid (15‐HETE).

## 3 RESULTS

**FIGURE 1:**
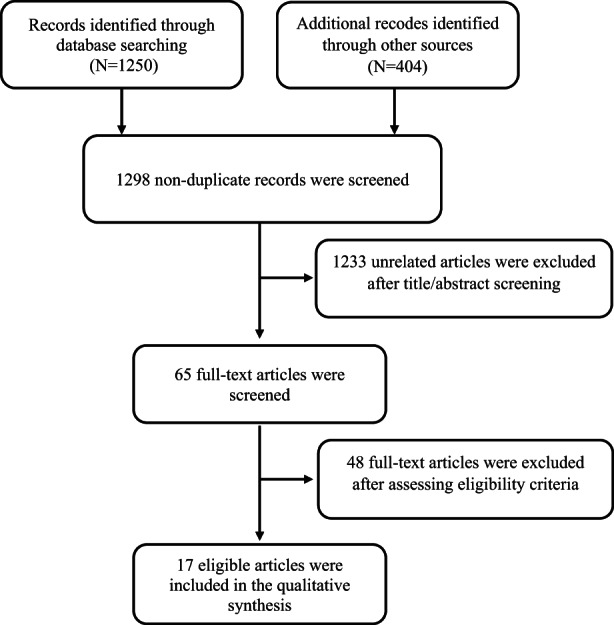
Flow diagram illustrating data collection protocol employed in this study.

We initially identified 1298 nonduplicate articles through a systematic search. After appraising the title/abstract of articles, 1233 articles were excluded due to not meeting inclusion criteria. Of the 65 remained articles, finally, 17 full‐text articles were selected that could gain inclusion criteria (Figure 1). Twelve papers have evaluated the effect of ginger or its derivatives on cell culture conditions, and five papers have evaluated the effect of ginger and its derivatives on colorectal cancer biomarkers in clinical trials.

### 3.1 Biomarker analyzed on in vitro study

Here are six articles that studied molecular biomarkers (Akimoto et al., 2015; Choudhury et al., 2010; Lee et al., 2008; Miyoshi et al., 2003; Ramachandran, Quirin, et al., 2015; Saha et al., 2014), that the molecular factors of Bax and Bcl2 studied widely. Studies showed that Bax is increased and induced the opening of the mitochondrial voltage‐dependent anion channel, so the death of the cell is guaranteed (Mignard et al., 2014). The Bcl‐2 family of regulator proteins regulated apoptosis, by either inhibiting (anti‐apoptotic) or inducing (pro‐apoptotic) apoptosis. In all of these studies, it has been shown that the amount of Bcl‐2 in the treated cells with ginger has decreased, and conversely, Bax has increased.

Seven articles studied the role of proliferative factors and MMPs (Lee et al., 2008; Ling et al., 2010; Ramachandran, Quirin, et al., 2015). MMPs have been implicated as possible mediators of invasion and metastasis in some cancers (Kim et al., 2014). Five studies have studied the anti‐inflammatory effects and these papers have shown that treating cells with ginger reduced inflammation (Ishiguro et al., 2007; Ling et al., 2010; Miyoshi et al., 2003; Romero et al., 2018; Saha et al., 2014). Also, four papers have proven that ginger has increased the activity of caspases (Choudhury et al., 2010; Ishiguro et al., 2007; Miyoshi et al., 2003; Ramachandran, Quirin, et al., 2015). All in vitro studies have shown that the treatment of cells with ginger stops their cell growth and causes cell death (Table 1).

**TABLE 1:** Effect of ginger extract on cell culture biomarkers

First author and contrary	Study design	Type of treatment	Culture media	Methodology	Dose of treatment	Duration	Events observed
Ramachandran et al. (2016) India	In vitro	Supercritical CO_2_ extract of mango ginger ± Irinotecan	(U87MG), human cell line	Genes associated with apoptosis, Compu Syn analysis, Xenograft.	50 μg/ml	72 h	↓Bcl‐2, mutant p53, (COX‐2) (CCNB2) genes. ↓MMP2, ↓MMP9, ↓TIMP1, ↓TIMP2, ↓VEGF, and ↓N‐myc. ↑p21, caspase‐3, inhibition of cell proliferation.
Lee et al. (2008), South Korea	In vitro	Chloroform extract of *Zingiber*	A549, human cell line	Cell cycle, Western blot analysis	5‐35 μM/L	72 h	Suppress cell cycle, ↑ p21 expression, ↓CDKs, inhibition of cell proliferation.
Rhode et al. (2007), USA	In vitro	Extraction of ethanol/water	SKOV3, A2780, ES2, CaOV3. Human cell lines	ELISA, plasmids, and immunoblotting	50, 75, 100 μg/ml	1, 3, 5 days	Ginger inhibits NF‐κB, ginger Inhibits IL‐8, and VEGF Secretion, inhibition of cell proliferation
Ling et al. (2010), Singapore	In vitro	6, 8, and 10‐shogaol	MCF‐7, MDA‐MB‐231. Human cell lines	Cell viability, Cell invasion, Gelatin zymography, (RT‐PCR), Western blot, nuclear fractions.	5–35 μM	24 h	Inhibitor effects on invasion of cells, ↓MMP‐9, ↓MMP‐7, and ↓MMP‐13, but MMP‐1, 3 have not decreased. Inhibition of NF‐kB signaling, inhibition of cell proliferation.
Ishiguro et al. (2007), Japan	In vitro	6‐gingerol + TRAIL	HGC, AGS, KATO III. Human cell lines	Tumor growth, Apoptosis, Caspase activity and Luciferase reporter assay, RT‐PCR, Immunocytochemical staining, and cell cycle	1 mg/ml	24 h	6‐gingerol+TRAIL led to the reduction of gastric cancer cells, inhibiting NF‐κB, ↑caspase‐3/7 activation, damages microtubules, inhibition of cell proliferation.
Choudhury et al. (2010), India	In vitro	Ginger aqueous extract	HeLa, A549 cells. Human cell lines	Cell cycle, DNA synthesis, apoptotic, Western blot, interphase microtubule, flow cytometric	100–250 μg/ml	24 h	↑p53, reduction of mitochondrial membrane potential, ↑Bax, ↓Bcl‐2, ↑Caspases, inhibition of tubulin polymerization, inhibition of cell proliferation
Akimoto et al. (2015), Japan	In vitro	[6]‐Shogaol and [6]‐gingerol	Anc‐1, AsPC‐1, BxPC‐3, CAPAN‐2, CFPAC‐1, MIAPaCa‐2 and SW1990. human cell lines	Cell cycle analysis and mitochondria membrane potential, apoptosis, western blotting, immunofluorescence	25–250 μg/ml	24 h	Inhibition of cell cycle. Increased the ratio of LC3‐II/LC3‐I, decreasing SQSTM1/p62 protein, inhibition of cell proliferation
Saha et al. (2014), USA	In vitro	6‐gingerol, 6‐Shogaol	LNCaP, DU145, and PC‐3. Human cell lines	Western blotting, apoptosis assay allograft tumor experiments	10,20,40 μM/L	24, 48, 72 h	↓ STAT3 and ↓NF‐kB, ↓ IL‐7, ↓CCL5, ↑Bax, ↓BCL2, ↑ p21, and ↑p27. Inhibition of cell proliferation
Park et al. (2014), South Korea	In vitro	Ginger methanol extract	HCT116, SW480, LoVo, MCF‐7, MDA‐MB231, HepG‐2 cells. Human cell lines	Western blot, RT‐PCR, transfection of small interference RNA (siRNA).	0–50–100‐200 mg/ml	24–48 h	↑ATF3, ERK1/2, and ATF. Inhibition of cell proliferation
Lee et al. (2008), South Korea	In vitro	[6]‐gingerol	MDA‐MB‐231. Human cell lines	Matrigel invasion assay, adhesion assay, MMP activity, RT‐PCR	2.5–10 μM/L	48 h	↓MMP‐2 or MMP‐9, ↓inhibits cell adhesion, invasion, motility, and activities MMP‐2 or MMP‐9. Inhibition of cell proliferation
Romero et al. (2018), Colombia	In vitro	Ginger ethanol extract	HT1080 cell. Human cell lines	Western blotting, Morphological assessment, ROS, Mitochondrial membrane potential quantification, H_2_O_2_ treatment	200–400 mg/ml	4 h	Decreasing ROS production, decreasing Mitochondrial membrane potential, ↓AKT activation, and inhibition of cell proliferation
Miyoshi et al. (2003), Japan	In vitro	[6]‐gingerol	human T‐lymphoma Jurkat cells	Immunoblot analysis, Measurement of mitochondrial transmembrane potential, Immunoblot analysis, cytochrome c release measurement, Analysis of DNA fragmentation	10–50 μM/L	6 h	↑caspase‐3 activation, ↑apoptosis, ↑ cytochrome c release, ↓Bcl‐2, ↑Bax, inhibition of cell proliferation

Abbreviations: 15‐PGDH, 15‐Hydroxyprostaglandin dehydrogenase; AA, arachidonic acid; CAT, catalase; COX, cyclooxygenase; GPx, glutathione peroxidase; GSH/GSSG, glutathione/glutathione disulfide; HETE, hydroxyeicosatetraenoic acid; HODE, hydroxyoctadecanoic acid; hTERT, human telomerase reverse transcriptase; LTB4, leukotriene B4; MDA, malondialdehyde; PGE_2_, prostaglandin E2, SOD, superoxide dismutase.

### 3.2 Biomarker analyzed on randomized clinical trials

Five clinical trials have studied the effect of ginger and its derivatives on colorectal cancer. Table 2 shows the quality of the studies based on the Cochrane risk‐of‐bias tool. Of these (Table 3), four have been conducted in the United States (Citronberg et al., 2013; Jiang et al., 2013; Zick et al., 2011; Zick et al., 2015). Another article from Thailand studied patients with colorectal cancer (Danwilai et al., 2017). The study by Danwilai et al., on 2017, subjects with colorectal cancer showed that the ginger significantly increased superoxide dismutase (837 ± 74.9 vs. 442.3 ± 31.6 U/ml), catalase (68.1 ± 3.4 vs. 44.4 ± 2.3 kU/ml), glutathione peroxidase (23.0 ± 1.3 vs. 10.8 ± 0.9 U/ml), and reduced/oxidized glutathione (23.4 ± 1.3 vs. 15.1 ± 1 U/ml) compared to placebo (Danwilai et al., 2017). In four articles that were in America (Citronberg et al., 2013; Jiang et al., 2013; Zick et al., 2011; Zick et al., 2015), special markers were studied after the administration of the same dose of ginger (2 g/day) for patients with colorectal cancer or high‐risk individuals. Citronberg et al., reported that consumption of 2 g/day ginger for 28 days unchanged the expression of Bax (15.6%; *p* = .78), p21 (18.2%; *p* = .43), Bcl2, and MIB‐1/Ki‐67 (16.9%; *p* = .39) in the whole colorectal crypts, while human telomerase reverse transcriptase (hTERT) expression significantly decreased in the whole colorectal crypts (41.2%; *p* = .05) and in the upper 40% of crypts (47.9%; *p* = .04) (Citronberg et al., 2013). Jiang et al. showed that ginger consumption significantly altered colonic COX‐1 protein level (−23.82 ± 41.76%) compared to placebo (18.86 ± 52.21%) in subjects at high risk for colorectal cancer (*p* = .03). There was no significant change in COX‐1 and 15‐hydroxyprostaglandin dehydrogenase (15‐PGDH) in subjects at normal risk for colorectal cancer (Jiang et al., 2013). Zick et al. reported that ginger intake had no significant effect on colonic PGE2, HETE‐5, HETE‐12, HETE‐15, and HODE‐13. But when these eicosanoids standardized to arachidonic acid, ginger significantly reduced colonic PGE2 (−28% vs. +26%, *p* = .05) and 5‐HETE (−15.8% vs. +26.7%; *p* = .04) as compared to placebo among subjects at normal risk for colorectal cancer (Zick et al., 2011). In another study, Zick et al. showed that ginger significantly increased colonic leukotriene B4 (LTB4) (54.0 ± 63.2% vs. −4.7 ± 54.9%; *p* = .04) and decreased arachidonic acid (−44.2 ± 41.5% vs. 229.4 ± 413.7%; *p* = .05) as compared to placebo among subjects at increased risk for colorectal cancer. There were no significant differences in colonic PGE2, HETE‐5, HETE‐12, HETE‐15, and HODE‐13. When these eicosanoids standardized to arachidonic acid, no effects were found (Zick et al., 2015).

**TABLE 2:** The quality of the clinical trial studies based on the Cochrane risk‐of‐bias tool

Study country/region	Sequence generation	Allocation concealment	Blinding of participants or personnel	Blinding of outcome assessment	Incomplete outcome data	Selective outcome reporting	Score	Quality
Jiang et al. (2013)	✓	?	✓	✓	✓	✓	**5**	**Good**
Zick et al. (2015)	✓	?	✓	✓	✓	✓	**5**	**Good**
Danwilai et al. (2017)	✓	?	✓	?	✓	✓	**4**	**Good**
Citronberg et al. (2013)	✓	?	✓	✓	✓	✓	**5**	**Good**
Zick et al. (2011)	✓	?	✓	✓	✓	✓	**5**	**Good**

**TABLE 3:** Effect of ginger extract on colorectal cancer biomarker

Country/region	Authors, years of publication	Sample size	Drug form and dosage	Time (day)	Previous treatment	Biomarkers	Assessment
United States	Jiang et al. (2013)	20	2 g, manufactured ginger extract	28	Not have taken aspirin or related NSAIDs, corticosteroid	COX‐1, 15‐PGDH	Protein level of COX‐1 reduced, protein levels of 15‐PGDH in the colon were unchanged
United States	Zick et al. (2015)	20	2 g, manufactured ginger extract	28	Non	AA, PGE2, LTB4, 5‐, 12‐, and 15‐HETE or 13‐HODE	↓AA, ↑ LTB4. Ginger lacks the ability to decrease eicosanoid levels in people at increased risk for colorectal cancer
Thailand	Danwilai et al. (2017)	43	2 g, standardized 6‐gingerol	3 days prior to chemotherapy	Chemotherapy	(SOD), (CAT), (GPx), (GSH/GSSG), (MDA), and NO2−/NO3−	In the ginger group SOD, CAT, GPx, and GSH/GSSG were significantly increased at day 64. while MDA and NO2−/NO3− levels were significantly decreased
United States	Citronberg et al. (2013)	20	2 g, manufactured ginger extract	28	Non	Measurement of Bax, Bcl‐2, p21, hTERT, and MIB‐1	p21 and Bcl‐2 expression unchanged, decreasing hTERT, MIB‐1 (Ki‐67), and Bax expression
United States	Zick et al. (2011)	20	2 g, manufactured ginger extract	28	Not have taken aspirin or related NSAIDs	AA, PGE2, 5‐, 12‐, and 15‐HETE or 13‐HODE	Ginger decreased COX and LOX‐5, 12‐, and 15‐2 enzymes as observed by significant or close to significant decreases in the mean percent change in PGE2, 5‐, 12‐, and 15‐HETE normalized to AA

Abbreviations: 15‐PGDH, 15‐Hydroxyprostaglandin dehydrogenase; AA, arachidonic acid; CAT, catalase; COX, cyclooxygenase; GPx, glutathione peroxidase; GSH/GSSG, glutathione/glutathione disulfide; HETE, hydroxyeicosatetraenoic acid; HODE, hydroxyoctadecanoic acid; hTERT, human telomerase reverse transcriptase; LTB4, leukotriene B4; MDA, malondialdehyde; PGE_2_, prostaglandin E2; SOD, superoxide dismutase.

From these clinical trials, only two clinical trials reported complications related to ginger consumption. Zick et al. reported gastrointestinal symptoms in five subjects (35.7%) and headache in one subject (7.14; discontinue) with ginger consumption among subjects at normal risk for colorectal cancer (Zick et al., 2011). In another study, the serum levels of alanine aminotransferase were significantly increased in two subjects (from 35 to 65 and 31 to 42 U/L) (Danwilai et al., 2017). No significant adverse events related to ginger extract were observed in both studies.

## 4 DISCUSSION

Apoptosis is programmed cell death that provides instructions and conditions for cancer cell death (Wlodkowic et al., 2011). Caspases are a family of protease enzymes and play essential roles in programmed cell death (Galluzzi et al., 2016). In vitro studies have shown that treatment with ginger stops cancer cell growth and causes cell death through the activation of Bax. This protein increases and induces the opening of the mitochondrial voltage‐dependent anion channel, so the death of the cell is guaranteed (Mignard et al., 2014). The Bcl‐2 is a family of proteins regulating apoptosis, by either inhibiting (anti‐apoptotic) or inducing (pro‐apoptotic) apoptosis. The in vitro studies have shown that the amount of Bcl‐2 in the treated cells with ginger has decreased, while Bax protein has increased. In addition, several in vitro studies examined the role of proliferative factors and MMPs (Choudhury et al., 2010; Ishiguro et al., 2007; Ling et al., 2010; Ramachandran, Quirin, et al., 2015; Rhode et al., 2007). MMPs have been implicated as possible mediators of invasion and metastasis in some cancers (Kim et al., 2014). Although different cell lines were used, this proves that in a variety of conditions, cell culture results in vitro models are similar and reliable but the heterogeneity of the study design is a limitation, which could make the studies difficult to compare. Also, there is a great variety of studies on different cancer cells. These data are an efficient tool for an indication of the anticancer role of ginger.

**FIGURE 2:**
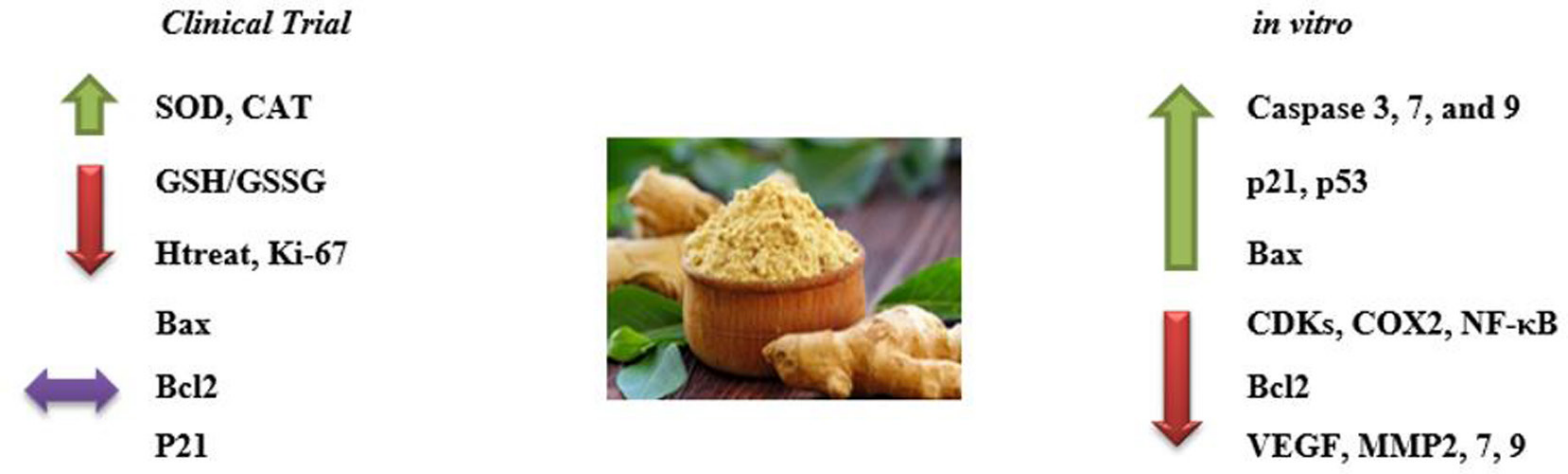
The effect of ginger on cancer cell biomarkers in clinical trials and cell culture. [down arrow (decrease); up arrow (increase); double‐sided arrow (without change)]. BAX, Bcl‐2 Associated X‐protein; Bcl‐2, B‐cell lymphoma 2; CAT: catalase; CDK, cyclin‐dependent kinases; COX2, cyclo‐oxygenase 2; GSH, glutathione; MMP, matrix metalloproteinases; NF‐κB, nuclear factor kappa‐B; SOD, superoxide dismutase; VEGF, vascular endothelial growth factor.

The results of in vitro studies showed that ginger and its derivatives have a high ability to induce apoptosis in a wide range of cancer cells (Figure 2). Now the question arises, are the results obtained under cell culture conditions transferable to clinical trials? Danwilai et al. showed that ginger significantly increased superoxide dismutase, catalase, glutathione peroxidase, and reduced/oxidized glutathione in people with colorectal cancer. An increase in all of these enzymes has been associated with a reduction in inflammation, which plays a vital role in cancer control. Ginger interferes with several cell‐signaling pathways that are important in the early development of cancer. In vitro studies showed that ginger has a powerful role in inhibiting COXs. These enzymes are responsible for the formation of thromboxane and prostaglandins from arachidonic acid (Ogunwobi et al., 2012). Nonsteroidal anti‐inflammatory drugs (NSAIDs) such as aspirin exert their effects through the inhibition of COX. One of the primary effectors of COX‐dependent mechanisms in carcinogenesis is likely to be prostaglandins in particular PGE2. This prostaglandin increased cellular proliferation, migration, and invasiveness, promotes angiogenesis, induces resistance to apoptosis, and modulates cellular and humoral immunity (Greenhough et al., 2009). Aspirin's most well‐characterized pharmacologic activity is the permanent modification of the COX enzymes (Patrono et al., 2005; Sankaranarayanan et al., 2020). There is also a non‐COX‐related pathway that might mediate aspirin's anticancer effects by suppressing nuclear factor kappa B (NF‐κB) expression (Fu et al., 2019).

Several studies revealed that ginger inhibited 5‐LOX, COX‐1, and COX‐2 (Rondanelli et al., 2020; Shukla & Singh, 2007). RCTs studied by Jiang et al., and M. Zick et al. showed that ginger decreased the amount of COX‐1 expression in the colonic mucosa of humans at increased risk of colorectal cancer. The ginger had an inhibitory effect on COX‐1, 5‐LOX, 12‐LOX, and 15‐LOX‐2 enzymes. Also, results showed a significant decrease in PGE‐2, PGE‐5, PGE‐12, and 15‐HETE normalized to arachidonic acid (Rhode et al., 2007). PGE‐2, as a product of COX, induces resistance to apoptosis and increases cellular proliferation. Danwilai et al. showed that ginger significantly increased SOD, CAT, GPx, and GSH/GSSG (Danwilai et al., 2017). These results confirm that ginger has anti‐inflammatory effects in patients with colorectal cancer (Rondanelli et al., 2020; Shukla & Singh, 2007).

To identify the more precise anticancer mechanisms of ginger, Citronberg et al. analyzed molecular pathways and key factors, including Bax, Bcl‐2, p21, hTERT, and MIB‐1 (Ki‐67) in colorectal crypts using automated immunohistochemistry and quantitative image analysis (Citronberg et al., 2013). The results were contrary to the results of in vitro studies. They showed that Bax increased in recipients of ginger, but results not show a change in Bcl2 and p21. However, p21 is a potent inducer of differentiation in intestinal colonocytes. It has been reported that the expression of Bax in the early stages of tumorigenesis in the colon is reduced, and abnormalities in p21 expression have been linked to carcinogenesis (Pryczynicz et al., 2014; Shukla & Singh, 2007). It should be noted that the studies were conducted on people at risk of colorectal cancer and not on people with colorectal cancer (Citronberg et al., 2013; Jiang et al., 2013; Zick et al., 2011; Zick et al., 2015). Somehow, these individuals do not have abnormal biomarkers and their biomarkers are in normal condition, in other words, if Bcl2 and p21 have not increased, also we should not expect molecular markers to change in these individuals. A remarkable point in these studies is that the amount of proliferative factors, such as hTERT and MIB‐1 (Ki‐67), has been significantly reduced. But the interesting tip is that ginger has been able to reduce hTERT and MIB‐1 (Ki‐67) in these individuals. A decrease in hTERT expression is consistent with previous reports, which found that ginger inhibited hTERT and c‐Myc expression in human lung cancer cells (Tuntiwechapikul et al., 2010). Oncogenes activate hTERT, while tumor suppressor p53 inhibits cancerous cell growth (Kyo et al., 2008). The MIB‐1 (Ki‐67) protein is a cellular marker for the proliferation of cells. It is strictly associated with cell proliferation (Cuylen et al., 2016). However, factor MIB‐1 (Ki‐67) decreased (Citronberg et al., 2013). One of the most important deficiencies in five clinical trials was the lack of clarification of the quantities of ginger derivatives in the blood. In other words, ginger derivatives have not been reported in the serum. The key point is that logically ginger may have the ability to activate or inhibit some of the pathways controlling cancer, and not all of them.

The main strength of this study was that the effect of ginger on cancer was compared both in cell culture conditions and in experimental conditions. In addition, this study was that each cancer marker was carefully analyzed in both conditions. One of the limitations of this study was that the number of clinical trial studies was not large and there is a need to investigate the effect of ginger on colorectal cancer in different places. Another limitation of this study was that the components of ginger in the serum of people were not investigated. If ginger compounds are measured in the serum of people, the effect of digestion and absorption on its consumption will be determined. Also, one of the significant drawbacks of the clinical trials studied was that none of these trials measured ginger derivatives in blood serum. Somehow, ginger and its derivatives are probably metabolized in the body so its effects are contradictory.

## 5 CONCLUSION

In vitro studies consistently showed the beneficial effect of ginger on the treatment and prevention of cancer cells in cell culture models, but the results of clinical trials do not justify these results. The results obtained from clinical trials have inconsistent. In part of clinical trial studies, the anticancer effect of ginger has been proven, and in the other part, this hypothesis has been rejected. This indicated that ginger may play a role in the prevention and treatment of cancer, but this is not a dominant role.

## CONFLICT OF INTEREST

The authors declare that they have no competing interests.

## Data Availability

Data sharing is not applicable to this article as no datasets were generated or analyzed during the current study.
